# 4,4′-Bipyridine–pyridine-3,5-dicarb­oxy­lic acid (3/4)

**DOI:** 10.1107/S160053681102719X

**Published:** 2011-07-13

**Authors:** Sheng-Bo Liu, Chao Xu, Taike Duan, Qun Chen, Qian-Feng Zhang

**Affiliations:** a Institute of Molecular Engineering and Applied Chemsitry, Anhui University of Technology, Ma’anshan, Anhui 243002, People’s Republic of China; b Department of Applied Chemistry, School of Petrochemical Engineering, Changzhou University, Jiangsu 213164, People’s Republic of China

## Abstract

In the title compound, 3C_10_H_8_N_2_·4C_7_H_5_NO_4_, the asymmetric unit contains two mol­ecules of pyridine-3,5-dicarb­oxy­lic acid and one mol­ecule of 4,4′-bipyridine in general positions together with one mol­ecule of 4,4′-bipyridine lying across a centre of inversion, thus giving a 4:3 molar ratio of pyridine-3,5-dicarb­oxy­lic acid to 4,4′-bipyridine. The dihedral angle between the bipyridine rings on general positions is 21.2 (2)°. These mol­ecular units are linked by O—H⋯N hydrogen bonds forming an extended two-dimensional framework in the crystal.

## Related literature

For structures and properties of self-assembled supermolecular compounds, see: Lehn (1990 ▶). For hydrogen bonds and π–π stacking inter­actions in supermolecular compounds, see: Roesky & Andruh (2003 ▶). For related structures, see: Soleimannejad *et al.* (2009 ▶); Jiang *et al.* (2010 ▶).
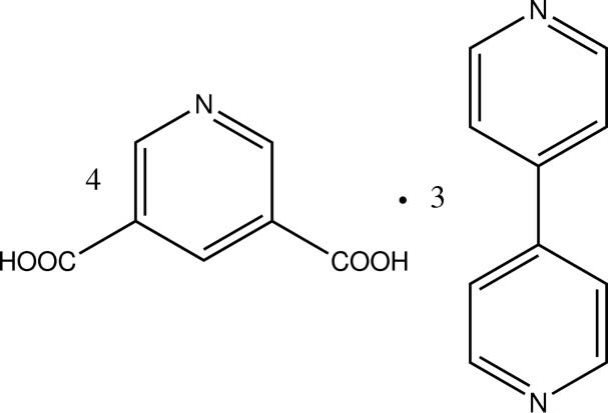


## Experimental

### 

#### Crystal data

3C_10_H_8_N_2_·4C_7_H_5_NO_4_
*M* *_r_* = 1137.04
Monoclinic, 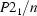

*a* = 13.8461 (4) Å
*b* = 11.0564 (3) Å
*c* = 18.1060 (4) Å
β = 110.511 (1)°
*V* = 2596.09 (12) Å^3^
*Z* = 2
Mo *K*α radiation
μ = 0.11 mm^−1^
*T* = 296 K
0.14 × 0.12 × 0.05 mm

#### Data collection

Bruker APEXII CCD area-detector diffractometer
Absorption correction: multi-scan (*SADABS*; Sheldrick, 1997 ▶) *T*_min_ = 0.985, *T*_max_ = 0.995
24265 measured reflections
5918 independent reflections
4086 reflections with *I* > 2σ(*I*)
*R*_int_ = 0.031

#### Refinement

*R*[*F* ^2^ > 2σ(*F* ^2^)] = 0.044
*wR*(*F* ^2^) = 0.118
*S* = 1.03
5918 reflections
379 parameters
H-atom parameters constrained
Δρ_max_ = 0.19 e Å^−3^
Δρ_min_ = −0.22 e Å^−3^

### 

Data collection: *APEX2* (Bruker, 2005 ▶); cell refinement: *SAINT* (Bruker, 2005 ▶); data reduction: *SAINT*; program(s) used to solve structure: *SHELXS97* (Sheldrick, 2008 ▶); program(s) used to refine structure: *SHELXL97* (Sheldrick, 2008 ▶); molecular graphics: *SHELXTL* (Sheldrick, 2008 ▶); software used to prepare material for publication: *SHELXTL*.

## Supplementary Material

Crystal structure: contains datablock(s) I, global. DOI: 10.1107/S160053681102719X/bx2358sup1.cif

Structure factors: contains datablock(s) I. DOI: 10.1107/S160053681102719X/bx2358Isup2.hkl

Supplementary material file. DOI: 10.1107/S160053681102719X/bx2358Isup3.cml

Additional supplementary materials:  crystallographic information; 3D view; checkCIF report

## Figures and Tables

**Table 1:** Hydrogen-bond geometry (Å, °)

*D*—H⋯*A*	*D*—H	H⋯*A*	*D*⋯*A*	*D*—H⋯*A*
O2—H2⋯N5^i^	0.82	1.80	2.5928 (17)	162
O4—H4⋯N4^ii^	0.82	1.79	2.6037 (18)	172
O5—H5⋯N3^iii^	0.82	1.78	2.5956 (17)	173
O7—H7⋯N2^ii^	0.82	1.83	2.5862 (16)	152

Symmetry codes: (i) 
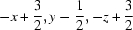
; (ii) 
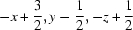
; (iii) 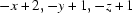.

**Fig. 1.**
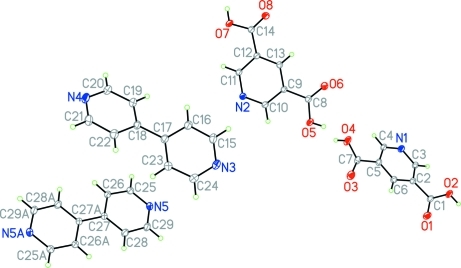
The structure of the title co-crystal compound, showing the atom-numbering scheme and displacement ellipsoids at the 50% probability level.

**Fig. 2.**
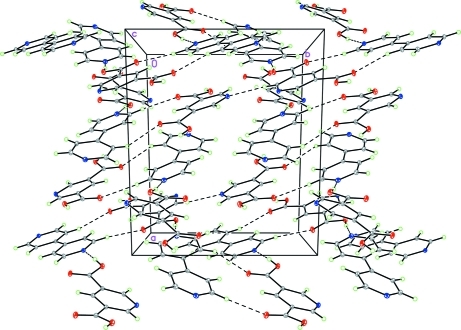
Packing diagram of the title compound viewed along b-axis showing hydrogen bonds as dash lines.

## supplementary crystallographic information

### Comment

In the past decades, the field of self-assembled supermolecular chemistry attracted increasing concerns due to their interesting structures and unique properties (Lehn, 1990). Hydrogen-bonds and pi—pi stacking are the most two important interactions in supermolecular compounds (Roesky & Andruh, 2003). As well known, many organic molecules with N– and O-donors such as bipyridine and pyridine-carboxylate acid have been wildly employed for constructing supermolecular complexes. In this paper, we report the hydrothermal synthesis and co-crystal structure of a two-dimensional framework of 4,4'-bipyridine (4,4'-bipy) and pyridine-3,5-dicarboxylic acid (3,5-pydcH_2_) assembled by the intermolecular hydrogen-bonding interactions. In the title compound, the asymmetric unit is formed by two molecules of pyridine-3,5-dicarboxylic acid and one molecule of 4,4'-bipyridine lying in general positions, together with one molecule of 4,4'-bipyridine lying across a centre of inversion, thus giving a 4:3 molar ratio of pyridine-3,5-dicarboxylic acid to 4,4'-bipyridine. These molecular units are linked by O—H···N hydrogen-bonds forming an extended two-dimensional framework, Table 1, Fig.2. The geometric parameters are in normal range.

### Experimental

4, 4'-bipyridine (31.2 mg, 0.2 mmol) and 3, 5-pyridinedicarboxylic acid (50.1 mg, 0.3 mmol) were mixed in 2.5 ml water solution and the mixture was stirred for a while. The solution was transferred to a 23 ml Teflon-lined stainless autoclave. The vessel was sealed and heated to 110 C° for 2 days. The autoclave was cooled to ambient temperature. Colorless flake crystals of title co-crystal compound were obtained and air dried. Yield: 43.2 mg, *ca* 67%. Anal. Calcd. for C_29_H_21_O_8_N_5_: C, 61.4; H, 3.71; N, 12.3%. Found: C, 61.1; H, 3.65; N, 12.1%.

### Refinement

H atoms were positioned geometrically and refined using a riding model C—H = 0.93 Å and O—H = 0.82 Å  with *U*_iso_(H) = 1.2 and 1.5 time for *U*_eq_(C) and hydroxyl groups, respectively.

### Crystal data

**Table:**

3C_10_H_8_N_2_·4C_7_H_5_NO_4_	*F*(000) = 1180
*M**_r_* = 1137.04	*D*_x_ = 1.455 Mg m^−^^3^
Monoclinic, *P*2_1_/*n*	Mo *K*α radiation, λ = 0.71073 Å
*a* = 13.8461 (4) Å	Cell parameters from 6026 reflections
*b* = 11.0564 (3) Å	θ = 2.3–27.4°
*c* = 18.1060 (4) Å	µ = 0.11 mm^−^^1^
β = 110.511 (1)°	*T* = 296 K
*V* = 2596.09 (12) Å^3^	Bar, colourless
*Z* = 2	0.14 × 0.12 × 0.05 mm

### Data collection

**Table:**

Bruker APEXII CCD area-detector diffractometer	5918 independent reflections
Radiation source: fine-focus sealed tube	4086 reflections with *I* > 2σ(*I*)
graphite	*R*_int_ = 0.031
phi and ω scans	θ_max_ = 27.5°, θ_min_ = 1.6°
Absorption correction: multi-scan (*SADABS*; Sheldrick, 1997)	*h* = −17→17
*T*_min_ = 0.985, *T*_max_ = 0.995	*k* = −14→14
24265 measured reflections	*l* = −19→23

### Refinement

**Table:**

Refinement on *F*^2^	Primary atom site location: structure-invariant direct methods
Least-squares matrix: full	Secondary atom site location: difference Fourier map
*R*[*F*^2^ > 2σ(*F*^2^)] = 0.044	Hydrogen site location: inferred from neighbouring sites
*wR*(*F*^2^) = 0.118	H-atom parameters constrained
*S* = 1.03	*w* = 1/[σ^2^(*F*_o_^2^) + (0.058*P*)^2^ + 0.3037*P*] where *P* = (*F*_o_^2^ + 2*F*_c_^2^)/3
5918 reflections	(Δ/σ)_max_ = 0.001
379 parameters	Δρ_max_ = 0.19 e Å^−^^3^
0 restraints	Δρ_min_ = −0.22 e Å^−^^3^

### Special details

**Table:**

Geometry. All e.s.d.'s (except the e.s.d. in the dihedral angle between two l.s. planes) are estimated using the full covariance matrix. The cell e.s.d.'s are taken into account individually in the estimation of e.s.d.'s in distances, angles and torsion angles; correlations between e.s.d.'s in cell parameters are only used when they are defined by crystal symmetry. An approximate (isotropic) treatment of cell e.s.d.'s is used for estimating e.s.d.'s involving l.s. planes.
Refinement. Refinement of *F*^2^ against ALL reflections. The weighted *R*-factor *wR* and goodness of fit *S* are based on *F*^2^, conventional *R*-factors *R* are based on *F*, with *F* set to zero for negative *F*^2^. The threshold expression of *F*^2^ > σ(*F*^2^) is used only for calculating *R*-factors(gt) *etc*. and is not relevant to the choice of reflections for refinement. *R*-factors based on *F*^2^ are statistically about twice as large as those based on *F*, and *R*- factors based on ALL data will be even larger.

### Fractional atomic coordinates and isotropic or equivalent isotropic displacement parameters (Å^2^)

**Table:**

	*x*	*y*	*z*	*U*_iso_*/*U*_eq_	
O1	0.89232 (10)	0.36614 (11)	1.13048 (7)	0.0566 (4)	
O2	0.91840 (11)	0.19752 (11)	1.20316 (7)	0.0556 (4)	
H2	0.9475	0.2439	1.2395	0.083*	
O3	0.74670 (12)	0.28184 (12)	0.82900 (7)	0.0623 (4)	
O4	0.71741 (11)	0.08686 (12)	0.79668 (7)	0.0619 (4)	
H4	0.7058	0.1124	0.7519	0.093*	
O5	0.87147 (10)	0.21216 (11)	0.58878 (6)	0.0513 (3)	
H5	0.8940	0.2100	0.6371	0.077*	
O6	0.91991 (11)	0.01904 (12)	0.60066 (7)	0.0624 (4)	
O7	0.79322 (9)	−0.10426 (10)	0.23327 (6)	0.0469 (3)	
H7	0.7969	−0.1689	0.2123	0.070*	
O8	0.84624 (11)	−0.21541 (11)	0.34327 (7)	0.0577 (4)	
N1	0.75488 (11)	−0.01589 (13)	1.02110 (8)	0.0448 (4)	
N2	0.76208 (10)	0.19983 (11)	0.34879 (7)	0.0357 (3)	
N3	1.06273 (12)	0.77515 (15)	0.25843 (8)	0.0504 (4)	
N4	0.83365 (12)	0.65049 (16)	−0.14945 (8)	0.0520 (4)	
N5	0.51891 (10)	0.82688 (12)	0.16936 (7)	0.0391 (3)	
C1	0.88290 (13)	0.25840 (16)	1.13706 (9)	0.0403 (4)	
C2	0.82884 (12)	0.17824 (14)	1.06824 (9)	0.0360 (4)	
C3	0.79903 (13)	0.06220 (15)	1.07946 (9)	0.0412 (4)	
H3	0.8105	0.0371	1.1309	0.049*	
C4	0.74003 (12)	0.02231 (15)	0.94810 (9)	0.0399 (4)	
H4A	0.7118	−0.0317	0.9067	0.048*	
C5	0.76425 (12)	0.13790 (15)	0.93021 (9)	0.0365 (4)	
C6	0.80869 (12)	0.21694 (15)	0.99183 (9)	0.0387 (4)	
H6	0.8249	0.2955	0.9819	0.046*	
C7	0.74147 (13)	0.17741 (17)	0.84653 (9)	0.0435 (4)	
C8	0.88248 (12)	0.10611 (15)	0.56092 (9)	0.0387 (4)	
C9	0.84316 (11)	0.10225 (13)	0.47271 (8)	0.0326 (3)	
C10	0.79689 (12)	0.20178 (14)	0.42774 (9)	0.0341 (3)	
H10	0.7897	0.2724	0.4533	0.041*	
C11	0.77338 (12)	0.09854 (14)	0.31231 (9)	0.0351 (4)	
H11	0.7507	0.0980	0.2575	0.042*	
C12	0.81732 (11)	−0.00551 (13)	0.35251 (8)	0.0333 (3)	
C13	0.85226 (12)	−0.00289 (14)	0.43401 (9)	0.0353 (4)	
H13	0.8818	−0.0715	0.4628	0.042*	
C14	0.82183 (12)	−0.11939 (14)	0.30945 (9)	0.0365 (4)	
C15	1.05291 (14)	0.66299 (18)	0.23018 (10)	0.0517 (5)	
H15	1.0778	0.5997	0.2656	0.062*	
C16	1.00790 (13)	0.63556 (17)	0.15141 (10)	0.0468 (4)	
H16	1.0007	0.5554	0.1348	0.056*	
C17	0.97328 (12)	0.72881 (15)	0.09689 (9)	0.0377 (4)	
C18	0.92417 (12)	0.70293 (16)	0.01128 (9)	0.0380 (4)	
C19	0.88180 (13)	0.58973 (16)	−0.01519 (9)	0.0444 (4)	
H19	0.8830	0.5294	0.0209	0.053*	
C20	0.83816 (14)	0.56738 (18)	−0.09498 (10)	0.0501 (5)	
H20	0.8107	0.4911	−0.1115	0.060*	
C21	0.87225 (15)	0.75957 (19)	−0.12476 (10)	0.0557 (5)	
H21	0.8684	0.8187	−0.1622	0.067*	
C22	0.91764 (14)	0.78877 (17)	−0.04623 (10)	0.0484 (4)	
H22	0.9439	0.8660	−0.0317	0.058*	
C23	0.98663 (14)	0.84544 (17)	0.12672 (10)	0.0505 (5)	
H23	0.9666	0.9110	0.0926	0.061*	
C24	1.02949 (15)	0.86454 (18)	0.20685 (11)	0.0553 (5)	
H24	1.0354	0.9435	0.2256	0.066*	
C25	0.48131 (13)	0.77880 (15)	0.09709 (9)	0.0407 (4)	
H25	0.4599	0.6985	0.0919	0.049*	
C26	0.47296 (12)	0.84300 (14)	0.02999 (9)	0.0376 (4)	
H26	0.4469	0.8057	−0.0191	0.045*	
C27	0.50350 (11)	0.96381 (14)	0.03536 (8)	0.0323 (3)	
C28	0.54147 (13)	1.01290 (15)	0.11086 (9)	0.0438 (4)	
H28	0.5624	1.0933	0.1180	0.053*	
C29	0.54790 (13)	0.94206 (16)	0.17488 (9)	0.0444 (4)	
H29	0.5740	0.9767	0.2248	0.053*	

### Atomic displacement parameters (Å^2^)

**Table:**

	*U*^11^	*U*^22^	*U*^33^	*U*^12^	*U*^13^	*U*^23^
O1	0.0855 (9)	0.0374 (7)	0.0412 (7)	−0.0112 (6)	0.0149 (6)	−0.0045 (6)
O2	0.0860 (9)	0.0441 (7)	0.0262 (6)	−0.0050 (6)	0.0065 (6)	−0.0053 (5)
O3	0.0973 (11)	0.0522 (9)	0.0348 (7)	−0.0113 (7)	0.0198 (7)	0.0069 (6)
O4	0.0973 (10)	0.0599 (9)	0.0235 (6)	−0.0126 (7)	0.0148 (6)	−0.0025 (6)
O5	0.0793 (9)	0.0441 (7)	0.0249 (6)	0.0074 (6)	0.0109 (6)	−0.0062 (5)
O6	0.1012 (10)	0.0483 (8)	0.0291 (6)	0.0213 (7)	0.0123 (6)	0.0055 (6)
O7	0.0818 (9)	0.0287 (6)	0.0277 (6)	−0.0007 (5)	0.0160 (6)	−0.0037 (5)
O8	0.0974 (10)	0.0319 (7)	0.0366 (7)	0.0155 (6)	0.0147 (7)	0.0028 (5)
N1	0.0630 (9)	0.0356 (8)	0.0315 (8)	−0.0053 (6)	0.0110 (7)	−0.0003 (6)
N2	0.0488 (8)	0.0287 (7)	0.0277 (7)	0.0007 (6)	0.0111 (6)	0.0008 (5)
N3	0.0596 (9)	0.0583 (10)	0.0293 (8)	0.0016 (8)	0.0105 (7)	−0.0065 (7)
N4	0.0595 (9)	0.0672 (11)	0.0277 (8)	0.0081 (8)	0.0129 (7)	−0.0009 (8)
N5	0.0472 (8)	0.0396 (8)	0.0284 (7)	0.0025 (6)	0.0104 (6)	0.0056 (6)
C1	0.0531 (10)	0.0393 (10)	0.0283 (8)	−0.0024 (8)	0.0141 (7)	−0.0041 (7)
C2	0.0427 (9)	0.0362 (9)	0.0284 (8)	0.0006 (7)	0.0114 (7)	−0.0014 (7)
C3	0.0583 (10)	0.0381 (9)	0.0255 (8)	0.0010 (8)	0.0125 (7)	0.0009 (7)
C4	0.0486 (9)	0.0390 (9)	0.0286 (8)	−0.0021 (7)	0.0092 (7)	−0.0044 (7)
C5	0.0425 (9)	0.0409 (9)	0.0250 (8)	−0.0014 (7)	0.0104 (7)	−0.0001 (7)
C6	0.0487 (9)	0.0363 (9)	0.0313 (9)	−0.0033 (7)	0.0141 (7)	0.0013 (7)
C7	0.0491 (10)	0.0525 (11)	0.0278 (9)	−0.0062 (8)	0.0119 (7)	0.0005 (8)
C8	0.0488 (9)	0.0392 (10)	0.0271 (8)	0.0020 (7)	0.0121 (7)	−0.0024 (7)
C9	0.0404 (8)	0.0315 (8)	0.0256 (8)	0.0001 (6)	0.0112 (6)	−0.0005 (6)
C10	0.0441 (9)	0.0297 (8)	0.0288 (8)	−0.0016 (7)	0.0131 (7)	−0.0032 (6)
C11	0.0473 (9)	0.0323 (8)	0.0241 (8)	−0.0019 (7)	0.0106 (6)	−0.0005 (6)
C12	0.0420 (8)	0.0290 (8)	0.0278 (8)	−0.0015 (6)	0.0110 (6)	−0.0020 (6)
C13	0.0464 (9)	0.0305 (8)	0.0267 (8)	0.0030 (7)	0.0100 (7)	0.0022 (6)
C14	0.0489 (9)	0.0304 (9)	0.0284 (8)	0.0010 (7)	0.0113 (7)	−0.0006 (6)
C15	0.0652 (12)	0.0530 (12)	0.0299 (9)	0.0009 (9)	0.0080 (8)	0.0027 (8)
C16	0.0588 (11)	0.0435 (10)	0.0340 (9)	−0.0013 (8)	0.0112 (8)	−0.0023 (8)
C17	0.0393 (8)	0.0444 (10)	0.0292 (8)	0.0030 (7)	0.0116 (7)	−0.0020 (7)
C18	0.0388 (8)	0.0477 (10)	0.0279 (8)	0.0066 (7)	0.0119 (7)	0.0006 (7)
C19	0.0513 (10)	0.0498 (11)	0.0298 (9)	0.0020 (8)	0.0114 (7)	0.0000 (7)
C20	0.0586 (11)	0.0560 (12)	0.0330 (9)	0.0013 (9)	0.0129 (8)	−0.0066 (8)
C21	0.0693 (13)	0.0634 (13)	0.0351 (10)	0.0069 (10)	0.0190 (9)	0.0098 (9)
C22	0.0583 (11)	0.0500 (11)	0.0370 (10)	0.0004 (8)	0.0166 (8)	0.0020 (8)
C23	0.0645 (11)	0.0435 (11)	0.0372 (10)	0.0082 (9)	0.0097 (8)	0.0006 (8)
C24	0.0693 (12)	0.0499 (11)	0.0409 (11)	0.0036 (9)	0.0119 (9)	−0.0121 (9)
C25	0.0529 (10)	0.0320 (9)	0.0353 (9)	−0.0008 (7)	0.0130 (8)	0.0026 (7)
C26	0.0496 (9)	0.0319 (9)	0.0276 (8)	−0.0005 (7)	0.0088 (7)	−0.0018 (7)
C27	0.0361 (8)	0.0317 (8)	0.0268 (8)	0.0015 (6)	0.0080 (6)	0.0016 (6)
C28	0.0607 (11)	0.0354 (9)	0.0306 (9)	−0.0111 (8)	0.0104 (8)	−0.0024 (7)
C29	0.0583 (10)	0.0442 (10)	0.0258 (8)	−0.0061 (8)	0.0086 (7)	−0.0013 (7)

### Geometric parameters (Å, °)

**Table:**

O1—C1	1.209 (2)	C9—C13	1.386 (2)
O2—C1	1.310 (2)	C10—H10	0.9300
O2—H2	0.8200	C11—C12	1.383 (2)
O3—C7	1.206 (2)	C11—H11	0.9300
O4—C7	1.311 (2)	C12—C13	1.383 (2)
O4—H4	0.8200	C12—C14	1.494 (2)
O5—C8	1.3063 (19)	C13—H13	0.9300
O5—H5	0.8200	C15—C16	1.375 (2)
O6—C8	1.2049 (19)	C15—H15	0.9300
O7—C14	1.3056 (18)	C16—C17	1.391 (2)
O7—H7	0.8200	C16—H16	0.9300
O8—C14	1.2129 (19)	C17—C23	1.385 (2)
N1—C4	1.333 (2)	C17—C18	1.486 (2)
N1—C3	1.335 (2)	C18—C22	1.388 (2)
N2—C11	1.3370 (19)	C18—C19	1.394 (2)
N2—C10	1.3391 (19)	C19—C20	1.379 (2)
N3—C24	1.326 (2)	C19—H19	0.9300
N3—C15	1.330 (2)	C20—H20	0.9300
N4—C21	1.331 (2)	C21—C22	1.376 (2)
N4—C20	1.333 (2)	C21—H21	0.9300
N5—C29	1.328 (2)	C22—H22	0.9300
N5—C25	1.337 (2)	C23—C24	1.378 (2)
C1—C2	1.498 (2)	C23—H23	0.9300
C2—C6	1.380 (2)	C24—H24	0.9300
C2—C3	1.384 (2)	C25—C26	1.377 (2)
C3—H3	0.9300	C25—H25	0.9300
C4—C5	1.388 (2)	C26—C27	1.394 (2)
C4—H4A	0.9300	C26—H26	0.9300
C5—C6	1.380 (2)	C27—C28	1.391 (2)
C5—C7	1.500 (2)	C27—C27^i^	1.484 (3)
C6—H6	0.9300	C28—C29	1.376 (2)
C8—C9	1.496 (2)	C28—H28	0.9300
C9—C10	1.385 (2)	C29—H29	0.9300
			
C1—O2—H2	109.5	O8—C14—O7	124.97 (15)
C7—O4—H4	109.5	O8—C14—C12	121.98 (14)
C8—O5—H5	109.5	O7—C14—C12	113.00 (13)
C14—O7—H7	109.5	N3—C15—C16	123.54 (17)
C4—N1—C3	116.65 (14)	N3—C15—H15	118.2
C11—N2—C10	118.70 (13)	C16—C15—H15	118.2
C24—N3—C15	117.42 (15)	C15—C16—C17	119.38 (17)
C21—N4—C20	117.81 (15)	C15—C16—H16	120.3
C29—N5—C25	117.28 (13)	C17—C16—H16	120.3
O1—C1—O2	125.29 (15)	C23—C17—C16	116.58 (15)
O1—C1—C2	122.74 (15)	C23—C17—C18	122.40 (15)
O2—C1—C2	111.97 (14)	C16—C17—C18	121.02 (15)
C6—C2—C3	117.94 (15)	C22—C18—C19	116.55 (15)
C6—C2—C1	121.12 (15)	C22—C18—C17	122.44 (16)
C3—C2—C1	120.93 (14)	C19—C18—C17	121.01 (15)
N1—C3—C2	124.09 (15)	C20—C19—C18	119.88 (17)
N1—C3—H3	118.0	C20—C19—H19	120.1
C2—C3—H3	118.0	C18—C19—H19	120.1
N1—C4—C5	123.85 (15)	N4—C20—C19	122.77 (18)
N1—C4—H4A	118.1	N4—C20—H20	118.6
C5—C4—H4A	118.1	C19—C20—H20	118.6
C6—C5—C4	118.04 (14)	N4—C21—C22	122.94 (17)
C6—C5—C7	120.54 (15)	N4—C21—H21	118.5
C4—C5—C7	121.41 (14)	C22—C21—H21	118.5
C2—C6—C5	119.35 (15)	C21—C22—C18	120.04 (18)
C2—C6—H6	120.3	C21—C22—H22	120.0
C5—C6—H6	120.3	C18—C22—H22	120.0
O3—C7—O4	125.04 (15)	C24—C23—C17	120.18 (17)
O3—C7—C5	122.25 (16)	C24—C23—H23	119.9
O4—C7—C5	112.70 (15)	C17—C23—H23	119.9
O6—C8—O5	124.79 (15)	N3—C24—C23	122.84 (18)
O6—C8—C9	122.28 (15)	N3—C24—H24	118.6
O5—C8—C9	112.93 (14)	C23—C24—H24	118.6
C10—C9—C13	118.32 (14)	N5—C25—C26	122.83 (15)
C10—C9—C8	121.67 (14)	N5—C25—H25	118.6
C13—C9—C8	120.00 (13)	C26—C25—H25	118.6
N2—C10—C9	122.28 (14)	C25—C26—C27	120.19 (14)
N2—C10—H10	118.9	C25—C26—H26	119.9
C9—C10—H10	118.9	C27—C26—H26	119.9
N2—C11—C12	122.87 (14)	C28—C27—C26	116.31 (14)
N2—C11—H11	118.6	C28—C27—C27^i^	121.69 (17)
C12—C11—H11	118.6	C26—C27—C27^i^	122.00 (17)
C13—C12—C11	117.97 (14)	C29—C28—C27	119.75 (15)
C13—C12—C14	120.78 (14)	C29—C28—H28	120.1
C11—C12—C14	121.17 (13)	C27—C28—H28	120.1
C12—C13—C9	119.84 (14)	N5—C29—C28	123.63 (15)
C12—C13—H13	120.1	N5—C29—H29	118.2
C9—C13—H13	120.1	C28—C29—H29	118.2

Symmetry codes: (i) −*x*+1, −*y*+2, −*z*.

### Hydrogen-bond geometry (Å, °)

**Table:**

*D*—H···*A*	*D*—H	H···*A*	*D*···*A*	*D*—H···*A*
O2—H2···N5^ii^	0.82	1.80	2.5928 (17)	162.
O4—H4···N4^iii^	0.82	1.79	2.6037 (18)	172.
O5—H5···N3^iv^	0.82	1.78	2.5956 (17)	173.
O7—H7···N2^iii^	0.82	1.83	2.5862 (16)	152.

Symmetry codes: (ii) −*x*+3/2, *y*−1/2, −*z*+3/2; (iii) −*x*+3/2, *y*−1/2, −*z*+1/2; (iv) −*x*+2, −*y*+1, −*z*+1.
